# CyAnimator: Simple Animations of Cytoscape Networks

**DOI:** 10.12688/f1000research.6852.2

**Published:** 2015-12-30

**Authors:** John H. Morris, Dhameliya Vijay, Steven Federowicz, Alexander R. Pico, Thomas E. Ferrin

**Affiliations:** 1 Resource for Biocomputing, Visualization and Informatics,, University of California, San Francisco, CA, 94143, USA; 2 Dhirubhai Ambani Institute of Information and Communication Technology, Gujarat, India; 3 Intrexon Inc., San Diego, CA, 92121, USA; 4 Gladstone Institutes, San Francisco, CA, USA; 5 Resource for Biocomputing, Visualization and Informatics, University of California, San Francisco, CA, USA

**Keywords:** Cytoscape, CyAnimator, network, animation

## Abstract

CyAnimator (http://apps.cytoscape.org/apps/cyanimator) is a Cytoscape app that provides a tool for simple animations of Cytoscape networks. The tool allows you to take a series of snapshots (CyAnimator calls them frames) of Cytoscape networks. For example, the first frame might be of a network shown from a ”zoomed out” viewpoint and the second frame might focus on a specific group of nodes. Once these two frames are captured by the tool, it can animate between them by interpolating the changes in location, zoom, node color, node size, edge thickness, presence or absence of annotations, etc. The animations may be saved as a series of individual frames, animated GIFs, or H.264/MP4 movies.

CyAnimator is available from within the Cytoscape App Manager or from the Cytoscape app store.

## Introduction

Biological networks are typically represented as nodes and edges (node-link diagrams) that might represent the pathways, signaling cascades, interactions between proteins, and other relationships between biological entities. The problem with this representation is that it makes it seem like these relationships are static, but it is well known that biological networks are dynamic, adapting and changing in response to the cell cycle, environmental conditions, development, and, over longer periods of time, evolution. One of the best methods to represent these changes is to take advantage of motion, showing changes by interpolating between the two states. This use of motion is one way to address “change blindness” ^1^, which makes it difficult to detect the difference between two images when they are shown in succession. This is important both for presentation of results to collaborators and the broader scientific community and for the exploration of data by individual researchers.

Cytoscape ^2^ is one of the most common tools used to visualize and analyze biological networks. It provides very powerful visualization tools to map a variety of categorical and numeric data into visual attributes associated with the nodes and edges of node-link diagrams. Unfortunately, Cytoscape does not provide any inherent animation capabilities, making it difficult to use interpolation to detect changes between two states, however, Cytoscape does provide a rich infrastructure for extending its core functionality through “apps” ^3^. Several Cytoscape apps provide some animation capabilities. For example, VistaClara ^4^, 3DScape ^5^, and clusterMaker ^6^ provide support to animate through the columns of a heat map. DynNetwork ( http://apps.cytoscape.org/apps/dynnetwork) reads specially constructed input files that specifically encodes changes to the network over time. Of these, only clusterMaker2 and DynNetwork are available in Cytoscape 3, and offer relatively limited restrictive animation capabilities (in the case of clusterMaker2) or require construction of special input files in advance (in the case of DynNetwork). None of the available tools allows for the animation of arbitrary changes to the network topology or visualization.

CyAnimator attempts to fill this gap by providing a tool that supports animation by allowing the user to designate particular network views as “key frames”. These frames represent the state of the network at particular moments in time including the current list of nodes and edges and the visual attributes of those nodes and edges. The user may then arrange those frames in a desired sequence and CyAnimator will interpolate between the frames resulting in a smooth animation between the states of the network. The resulting animation may be saved as a movie.

## Methods

### Implementation

The main object in CyAnimator is a CyFrame, which contains all of the information about the visual attributes of the network background, annotations, nodes, and edges. This information is stored in a series of maps indexed by the network, node, edge or annotation object. CyAnimator makes heavy use of the visual property system in Cytoscape and the pseudocode for the general loop for populating a CyFrame is:



                        getNetworkVisualProperties();
foreach Node:
   getNodeVisualProperties();
foreach Edge:
   getEdgeVisualProperties();
foreach Annotation:
   getAnnotationVisualProperties();
getImage();
					


The captured image is used to show thumbnails to the user in the CyAnimator dialog (see below). The result of the above pseudocode is the creation of 4 maps:



                        Map<CyNode, Map<VisualProperty<?>, Object>> nodePropertyMap;
Map<CyEdge, Map<VisualProperty<?>, Object>> edgePropertyMap;
Map<CyNetwork, Map<VisualProperty<?>, Object>> networkPropertyMap;
Map<Annotation, Map<VisualProperty<?>, Object>> annotationPropertyMap;
					


When the user clicks on a frame in the CyAnimator dialog or during the animation, the stored visual properties are mapped onto the current network. This is a straightforward mapping except in circumstances where the current network does not have the node, edge, or annotation that the stored CyFrame had or the CyFrame did not have a node, edge, or annotation that the current network has. In the first case, the missing object is added to the network and styled using the stored information. This approach depends on the fact that deleted nodes and edges are not removed from the CyRootNetwork (the root of the network collection), so the topology of the network at the time the CyFrame was stored may be recreated. In the second case, the CyFrame sets the visibility of the object to **false**, hiding that object, and resulting in a network view that is consistent with the saved visual properties.

Interpolation between CyFrames is handled by the Interpolator, which maintains lists of interpolators for node visual attributes, edge visual attributes, annotation visual attributes, and network visual attributes. The Interpolator *makeFrames*() method takes as input the list of CyFrames, the user saved (key frames) and returns an array of frames that includes interpolations between each pair of key frames. So, if the user has requested 30 frames between each key frame, and created 5 key frames, *makeFrames*(), will return 121 frames, which is the number of key frames to interpolate between (key frames - 1), multiplied by the number of interpolations between each key frame, plus the initial key frame ((keyframes - 1)*interpolations + 1).

CyAnimator currently supports several different interpolators:

**Table:** 

**Color**	Colors are linearly interpolated between the two key frames. Color interpolation may include interpolation of transparency using a Bezier to provide more natural fade-in/fade-out
**Size**	Linear interpolation of numerical values (usually sizes) between the two key frames.
**Transparency**	Linear interpolation of transparency values
**ObjectPosition**	Special interpolator for changes in label and custom graphics positions. Doesn’t support changes in justification or object anchor.
**Position**	Linearly interpolate changes in X,Y position of objects
**Crossfade**	For properties that can not be easily interpolated (e.g. node shape), fades the object out, then fades it in with the new property
**CustomGraphicsCrossfade**	Crossfade between two custom graphics.
**Visible**	Fades an object in or out if the visibility changes
**None**	This visual attribute is not interpolated.

Table 1 shows the interpolated visual attributes and the type of interpolation.

**Table 1.** Visual Property Interpolations.

Object Type	Visual Attributes	Interpolation
Network	Background Paint	**Color**
	X, Y, and Z Location	**Position**
	Depth, Height, Scale Factor, Size, and Width	**Size**
Node	Border Paint, Fill Color, Label Color	**Color**
	Border Transparency, Label Transparency, Transparency	**Transparency**
	Border Width, Height, Width, Size, Label Size, Custom Graphics Size (1–9)	**Size**
	X, Y, and Z Location	**Position**
	Shape, Label, Font Face,	**CrossFade**
	Label Position, Custom Graphics Position (1-9)	**ObjectPosition**
	Custom Graphics (1–9)	**CustomGraphicsCrossfade**
	Nested Network Visible	**None**
	Visible	**Visible**
Edge	Label Color, Paint	**Color**
	Label, Font Face, Line Type,	**Crossfade**
	Label Transparency, Transparency	**Transparency**
	Width, Label Size	**Size**
	Visible	**Visible**
Annotations	X and Y Location	**Position**
	Zoom, Width, Height, Border Width, Font Size, Image Contrast, Image Brightness, Arrow Source Size, Arrow Width, Arrow Target Size	**Size**
	Color, Font Color, Border Color, Arrow Source Color, Arrow Target Color, Arrow Color	**Color**
	Opacity, Image Opacity	**Transparency**
	Shape, Text, Font Style, Font Family, Image URL, Arrow Source Anchor, Arrow Target Anchor, Arrow Source Type, Arrow Target Type	**Crossfade**
	Canvas	**None**

To implement the creation of a movie, three different mechanisms are used. In each case, an image file is created for each interpolated frame. One option the user has is to just write out the individual frames and allow them to use whatever movie making tool they desire. We have also implemented an animated GIF writer using the native Java ImageIO library. Finally, for creating MP4 movies with the H.264 codec, we utilize the JCodec ( http://jcodec.org/) Java library, which includes the necessary video codecs.

CyAnimator is not able to handle all visual properties, however. Currently edge bends are not correctly interpolated and neither are certain changes in label position (particularly those that require knowledge of the rendered width or height). Furthermore, with the introduction of multiple back-end renderers in Cytoscape 3.2 and 3.3, it’s not clear how well CyAnimator will work with other renderer implementations.

### Operation

**Figure 1.**
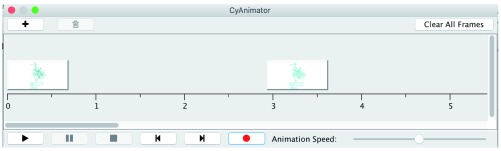
The CyAnimator Dialog showing two key frames. To support creating and modifying complicated movies, CyAnimator saves all of the frames as part of the session. When you open a Cytoscape session that has been saved with an active CyAnimator timeline, that timeline will be presented when the CyAnimator dialog is presented (Apps→CyAnimator).

To bring up CyAnimator select **Apps→CyAnimator**. This will bring up an empty CyAnimator dialog ( Figure 1). Note that CyAnimator is only able to animate between networks in the same network collection (In Cytoscape 3, a Network Collection contains multiple networks which possibly share nodes and edges). Starting CyAnimator on a new network collection will create a new, empty, CyAnimator dialog. Once a CyAnimator dialog is open, the general workflow is to manipulate the network to what you want it to look like at the start of your movie, then select 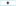 to add the frame to CyAnimator. Once the frame has been added, modify your network to what you want it to look like in the next frame of your movie and then again select 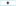. Note that CyAnimator will do all of the interpolation to get from one frame to another, so the manipulations of the network can include a variety of changes, including changes in color, position, zoom, annotations, etc. Repeat this process until you are happy with your movie, then simply press the record ( 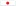) button. If you want more time between any two frames, simply drag the image. The timeline is measured in seconds and assumes that 30 frames will be interpolated for each second.

### CyAnimator dialog

The CyAnimator dialog provides the main interface to CyAnimator, including the following controls:

**Table:** 

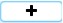	Add the current network view as a frame to the animation.
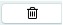	Remove all currently selected frames
**Clear All Frames**	Remove all frames from the timeline
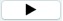	Play the animation by interpolating through each frame.
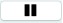	Pause the currently playing animation.
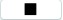	Stop the currently playing animation.
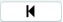	Step backwards to the previous interpolated frame.
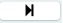	Step forwards to the next interpolated frame.
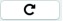	This toggle button controls whether the animation is looped or not. When the button is in, the animation will continue to loop, otherwise, it will stop at the end.
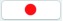	Bring up the Output Options dialog and record a movie or (optionally) save each of the interpolated frames.
**Animation Speed**	The speed slider controls the speed of the animation (but is ignored for the recorded movies, which uses it’s own Frames Per Second option.

Clicking on a frame and then dragging it will move the frame in the timeline. Holding down the shift key when clicking and dragging a frame will move all of the frames to the right the same amount. To make a frame current (i.e. show that frame in the Network Window), double-click on the frame. To delete a frame, click on the frame to select it, and press the trash can icon ( 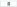).

### Output options

**Figure 2.**
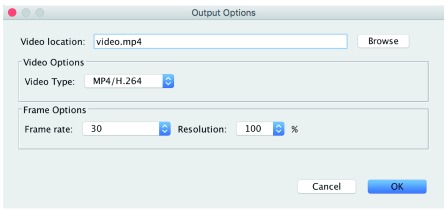
The Output Options Dialog showing the production of an MP4/H.264 video file at the screen resolution and 30 frames/second.

The Output Options Dialog (see Figure 2) provides the controls to choose the type of video you want to produce, options about the resolution and speed of the video, and the location of the video output.

### Video types

***Frames***. This is the simplest of the video types. This will output each frame as a ".png" file at the requested resolution (see ’Resolution’ below) into the Video location directory specified. To make a movie, you could use any of the standard video packages that accept individual frames (e.g. iMovie on Mac). Note that the Frames Per Second option doesn’t make sense for this output type since we’re only writing individual frames (no time encoding), so this option is disabled (grayed out) in the interface.

***GIF***. This will output an animated GIF of the interpolated frames. Animated GIF files are easy to show on web sites, but are not the currently accepted standard format. On the other hand, animated GIF files are computationally very easy to produce.

***MP4/H.264***. This will output a raw MPEG4 file of the interpolated frames. Producing an MP4 can be computationally expensive, and will require more time and memory to complete.

### Frame options

***Frame rate***. This controls the speed of the movie in terms of the number of frames per second. Four frame rates are offered, the two standard frame rates: 25 (PAL) and 29.97 (NTSC), and 30 and 60. The timeline dialog is calibrated at 30 frames per second, so a frame rate of 30 will correspond to the timeline and is a good default unless there is a reason to use one of the standards.

***Resolution***. This controls the resolution of the output frames. The units are % expansion, so a Resolution of 300 would result in a 300% (or 3X) expansion of the output image. This is extremely important for high-quality videos. We recommend at least a 2X (Resolution of 200) expansion for any published video.

### Example movie

A sample movie is provided (see Supplementary_File_1.mov) that was created with CyAnimator 2.0.2 and Cytoscape 3.3.0. This movie used galFiltered.cys, which may be located in the *sampleData* subdirectory of the Cytoscape installation directory (e.g. /Applications/Cytoscape_v3.3.0/sampleData on a Mac). The movie consisted of 13 key frames:

1. the full network using the default style
2. a view focused on MCM1 (YMR043W)
3. that same view using COMMON NAME for the node label and circle for node shape
4. same view as 3, but scaled the node size and node label size by Degree
5. added in some annotations to highlight MCM1
6. Change the node colors using the data from the gal1RGexp column. Color gradient is from blue to yellow. Added a text annotation describing the data
7. the same view as the above – to hold it on the screen
8. The same as #6, but using gal4RGexp as the column
9. Again, use the same frame before to provide some time onscreen
10. The same as #8, but using gal80Gexp as the column
11. Another frame of gal80Gexp
12. This frame uses the custom graphics Chart feature to show all three expression values and shows how custom graphics fade in. The labels were also moved down to avoid the charts
13. another capture frame of the same frame as #12

## Conclusions

CyAnimator is an important addition to the suite of Cytoscape apps. It provides an easy tool to interpolate between different states of a network and may be used to animate changes over time, condition, or treatment. In the future, we will be adding support for visualization engines other than current default, including the ability to animate 3D renderers.

## Software availability

### Software available from

http://apps.cytoscape.org/apps/cyanimator

### Latest source code

https://github.com/RBVI/CyAnimator

### Archived source code as at the time of publication

http://dx.doi.org/10.5281/zenodo.35386 ^7^

### License: Lesser GNU Public License 3.0

https://www.gnu.org/licenses/lgpl.html

## Supplementary material

**Sample movie created with CyAnimator and Cytoscape 3.3.** This movie used galFiltered.cys, which may be located in the sampleData subdirectory of the Cytoscape installation directory (e.g. /Applications/Cytoscape_v3.3.0/sampleData on a Mac) as a starting point.

.

See below for the saved session file.

http://dx.doi.org/10.5256/f1000research.6852.s110143

.

Session file used to create the sample video.

The session file saved with the timeline used to create the sample video.

http://dx.doi.org/10.5256/f1000research.6852.s110145
