# Supplementary material for: CyAnimator: Simple Animations of Cytoscape Networks
Source: F1000Res. 2015 Dec 30;4:482. Originally published 2015 Aug 5. [Version 2] doi: 10.12688/f1000research.6852.2 (PMC4752027; doi:10.12688/f1000research.6852.2)
Supplement: Peer review history

# Peer review: Referee response for version 2

**DOI:** 10.5256/f1000research.8183.r12291

**Published:** 2016-02-11

**Authors:** Piet Molenaar

**Affiliations:** 1 Academic Medical Center, Amsterdam, Netherlands

The CyAnimator app is a valuable addition to the Cytoscape network visualization tool. Cytoscape itself doesn't provide animation capabilities and the authors rightfully state that animation can provide additional insights for researchers.

There is some flickering visible while animating (nodes appearing and disappearing behind other nodes) but this apparently has to do with the current rendering engine. In future implementations it is expected that these issues will be solved.

I have read this submission. I believe that I have an appropriate level of expertise to confirm that it is of an acceptable scientific standard.

# Peer review: Referee response for version 1

**DOI:** 10.5256/f1000research.7370.r10174

**Published:** 2015-09-02

**Authors:** Nathan Salomonis

**Affiliations:** 1 Cincinnati Children's Hospital Medical Center Research Foundation, Cincinnati, OH, USA

The authors present a useful Cytoscape app that fills in an important void not provided by the parent application: dynamically transitioning between distinct network states or associated visualized datasets. The application is thus novel and provides an important function in Cytoscape that could significantly assist with biological interpretation.

**Major Point 1: **The authors state: “Note that CyAnimator is only able to animate between networks in the same network collection.”

Can a network collection be different networks that share some or all of the same nodes? If not, I understand how this could be a technical limitation of the app, but would be of particular use when curating distinct pathway states from an original network. Often, adding, subtracting, moving and re-coloring nodes in succession to different states in the same network instances is challenging, but storing these as distinct snap shots that can be animated together is extremely powerful. If not present, is this a future option?

**Major Point 2:** The shown movie is a reasonable simple demonstration of CyAnimator, however, this does not show of the more interesting dynamic options of the tools, such as transitioning between different developmental or temporal states (node color attributes, aka gene expression fold differences) or temporally distinct interactions for a given pathway (e.g., chain of metabolic events). While I realize this would be some work to do, this would more strongly demonstrate the capabilities of the software.

****

**Minor Point:** One of the powerful uses of this software would be via programmatic generation of animations, by defining network states and visual parameters. While I know that some of these capabilities are embedded in Cytoscape 3, are these possible within CyAnimator for the purpose of making animations outside of the UI? If so, can the authors provide an example set of code to do this? A link to documentation or a tutorial would also be helpful for the end-users to properly use the software.

I have read this submission. I believe that I have an appropriate level of expertise to confirm that it is of an acceptable scientific standard, however I have significant reservations, as outlined above.

# Response

**Published:** 2015-12-14

**Authors:** Scooter Morris

Thank you for your review.  I have just submitted version 2 of the CyAnimator paper, which corresponds to the latest release of the App on the Cytoscape App store.  The new app version is a significant rewrite and offers several advantages over the previous version, which I believe address move of your concerns.  To answer point-by-point:

***Major Point 1: **The authors state: “Note that CyAnimator is only able to animate between networks in the same network collection.”*

**

*Can a network collection be different networks that share some or all of the same nodes? If not, I understand how this could be a technical limitation of the app, but would be of particular use when curating distinct pathway states from an original network. Often, adding, subtracting, moving and re-coloring nodes in succession to different states in the same network instances is challenging, but storing these as distinct snap shots that can be animated together is extremely powerful. If not present, is this a future option?*

Yes, a network collection in Cytoscape 3 includes multiple networks that share nodes and edges.  Essentially, a network collection can be thought of as a set of projections of a single network, very similar to the Cytoscape 2 model.  Cytoscape 3 allows users to create multiple network collections and the nodes and edges aren't shared between them.  Both versions of the App support this capability.

***Major Point 2**: The shown movie is a reasonable simple demonstration of CyAnimator, however, this does not show of the more interesting dynamic options of the tools, such as transitioning between different developmental or temporal states (node color attributes, aka gene expression fold differences) or temporally distinct interactions for a given pathway (e.g., chain of metabolic events). While I realize this would be some work to do, this would more strongly demonstrate the capabilities of the software.*

The sample movie has been updated as suggested to show three different expression fold changes and a heat strip representation showing all three at once.

***Minor Point:** One of the powerful uses of this software would be via programmatic generation of animations, by defining network states and visual parameters. While I know that some of these capabilities are embedded in Cytoscape 3, are these possible within CyAnimator for the purpose of making animations outside of the UI? If so, can the authors provide an example set of code to do this? A link to documentation or a tutorial would also be helpful for the end-users to properly use the software.*

This version of the App introduces some simple commands that may be used by app developers to create frames.  At this point, the capability remains somewhat rudimentary and doesn't allow programmatic changes to frame ordering or transition duration.  This will be added in a future version and more detailed documentation will be written at that time.

# Peer review: Referee response for version 1

**DOI:** 10.5256/f1000research.7370.r9880

**Published:** 2015-08-18

**Authors:** Giovanni Scardoni

**Affiliations:** 1 Center for BioMedical Computing, University of Verona, Verona, Italy

The App is very useful and easy to use. As the article highlights It is very important to have a tool allowing the user to create animations from static frames of a network, and there are no other Cytoscape apps with the same characteristics  of CyAnimator. The paper is well written and both implementation and features are easy to understand.

Minor changes:

- The sentence about the future implementation of the maps in the implementation section should be moved to the conclusions session or to the supplementary materials. So the reader can concentrate on the current features of the app.
- Similarly, the last sentence of the conclusions sounds better as "The ability to save the key frames of an animation as part of a session will be added .....".

I have read this submission. I believe that I have an appropriate level of expertise to confirm that it is of an acceptable scientific standard.
